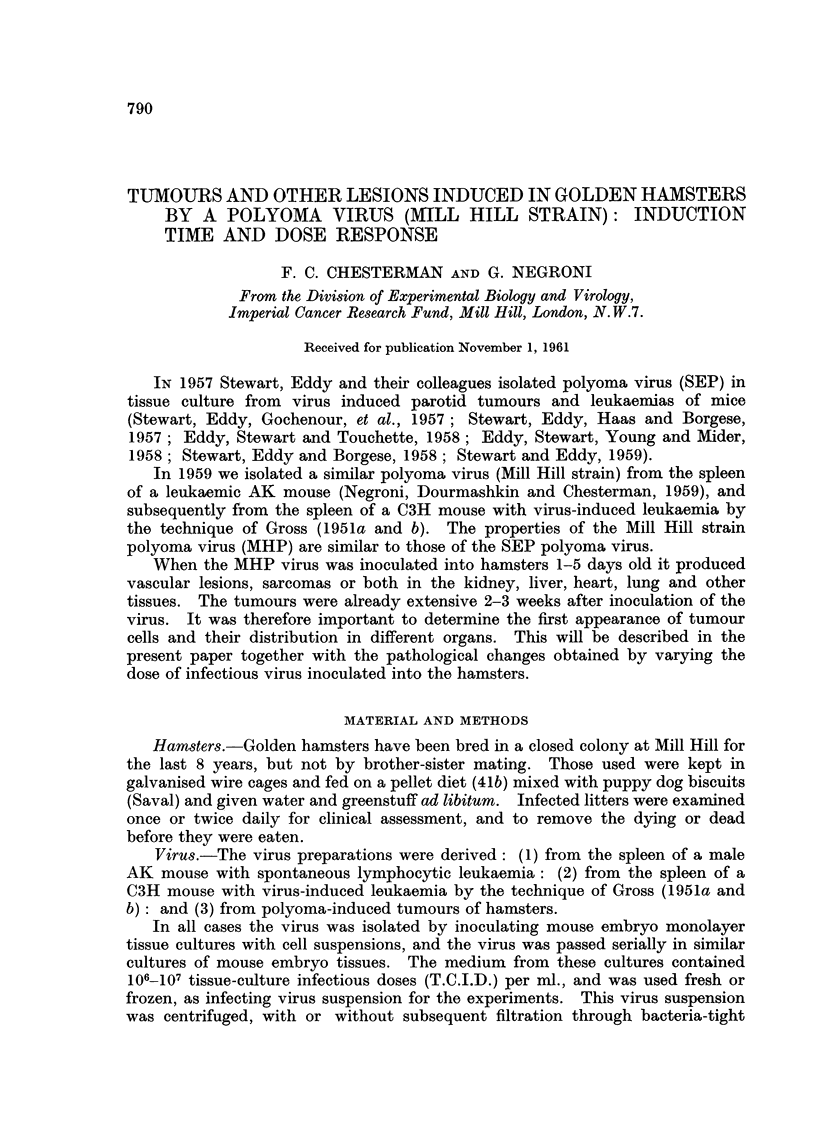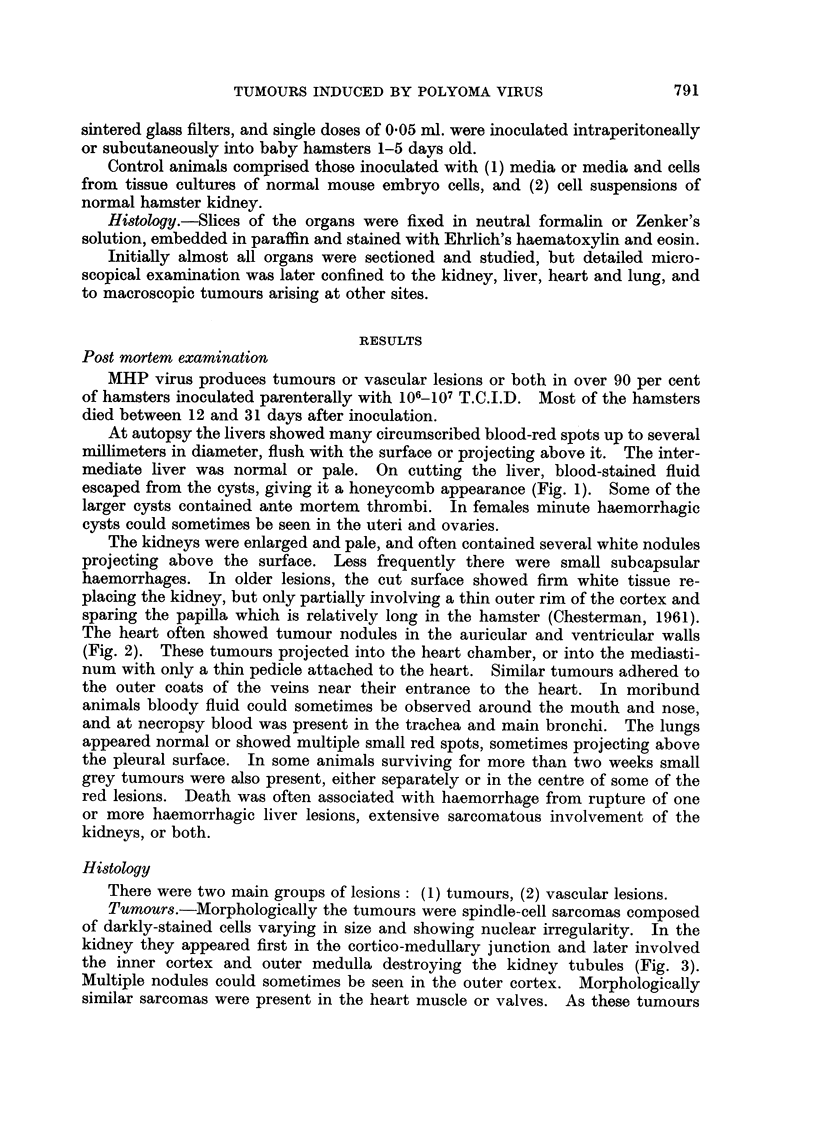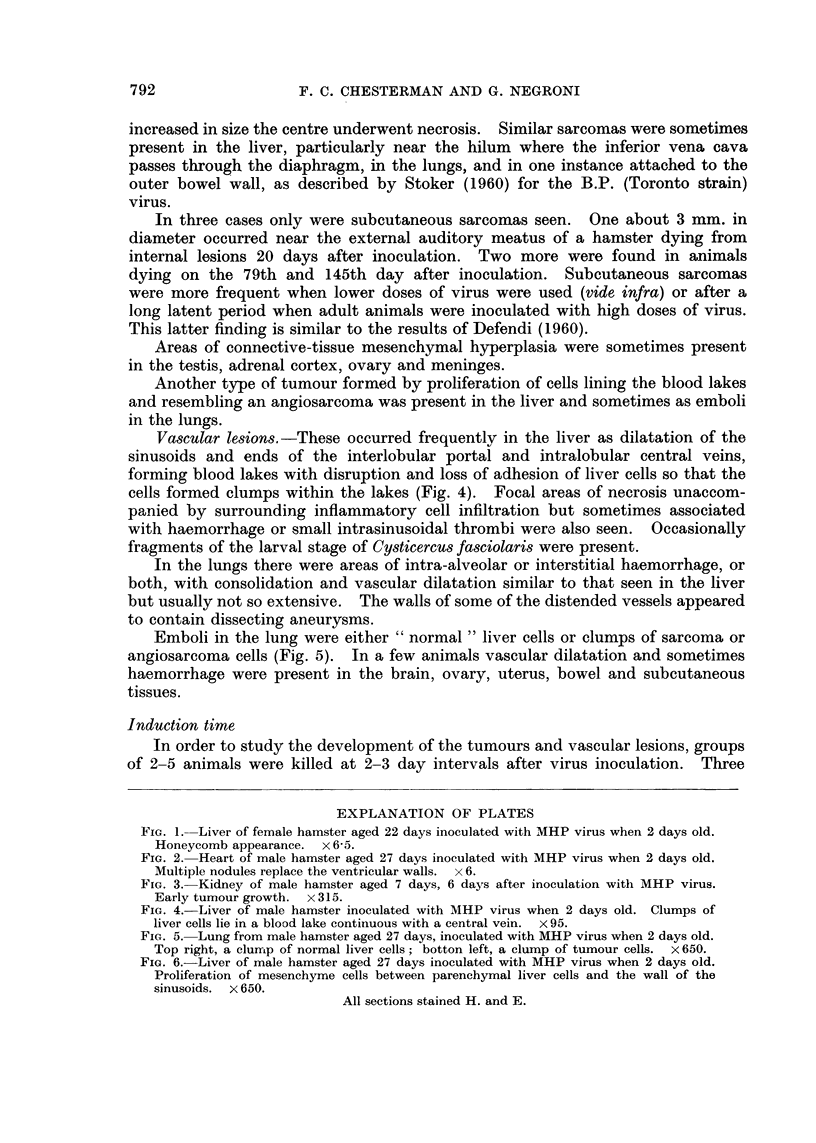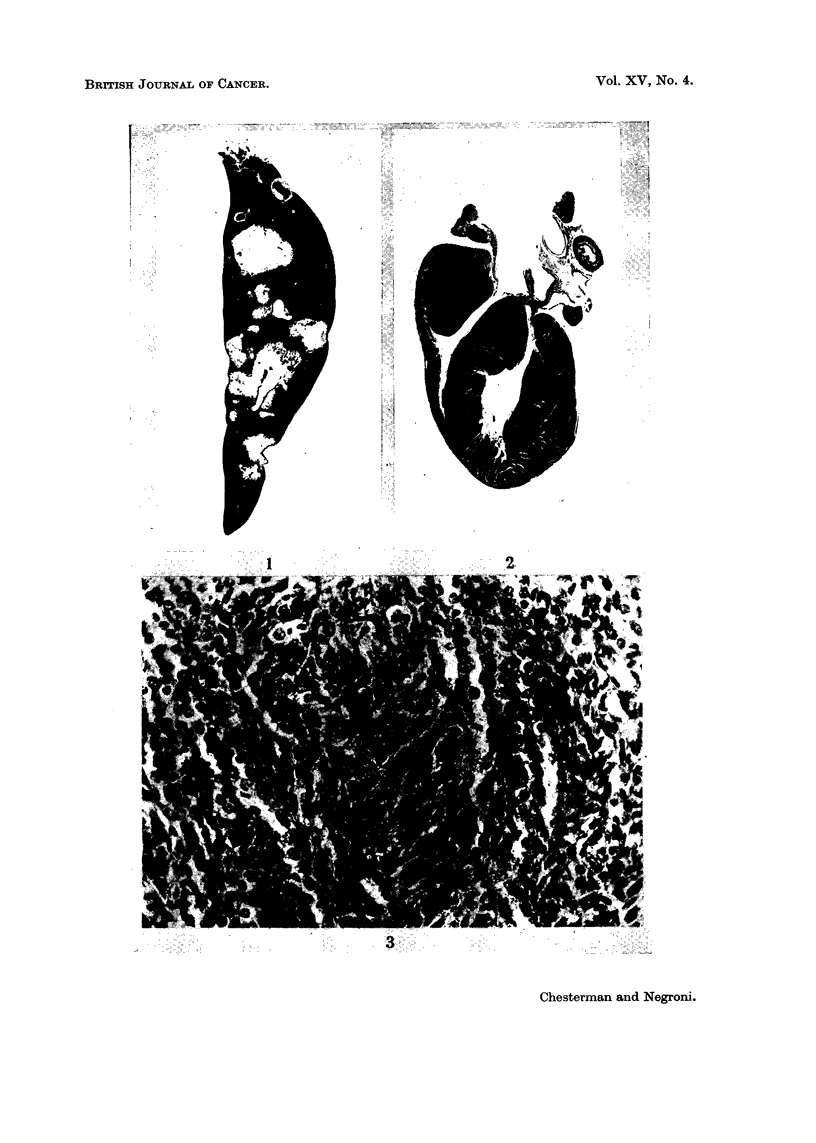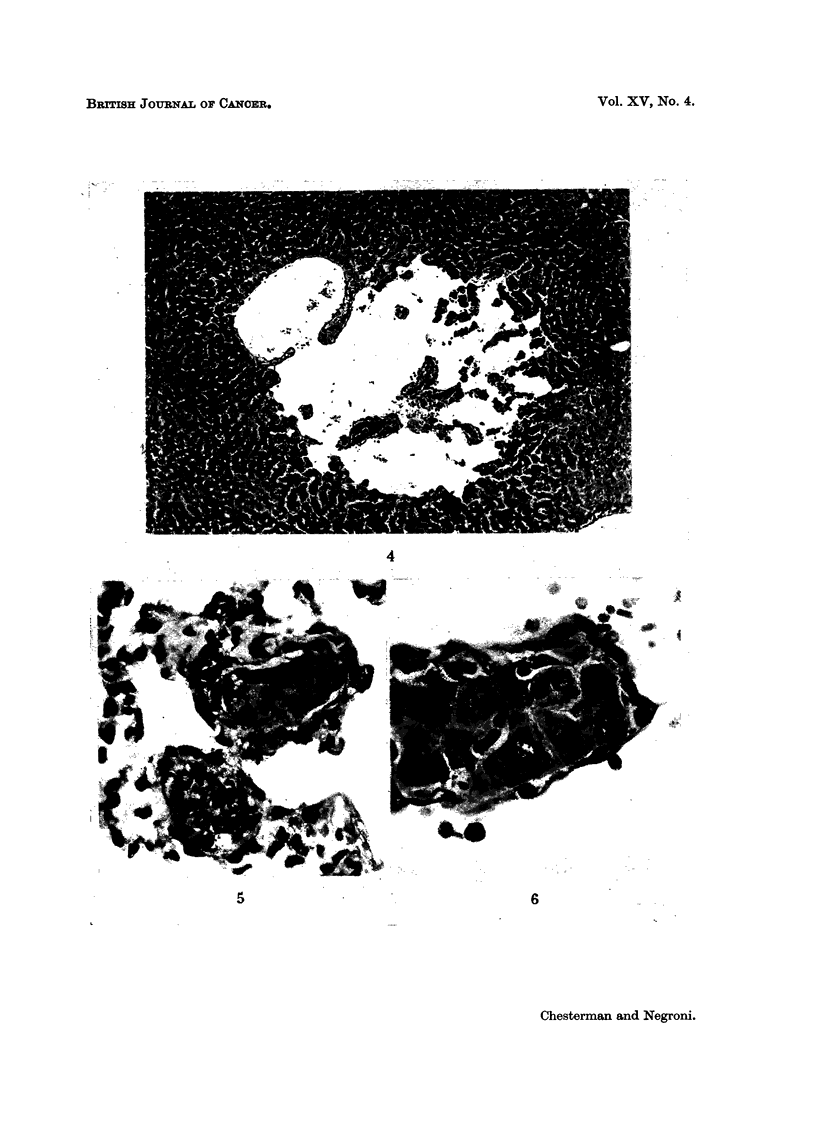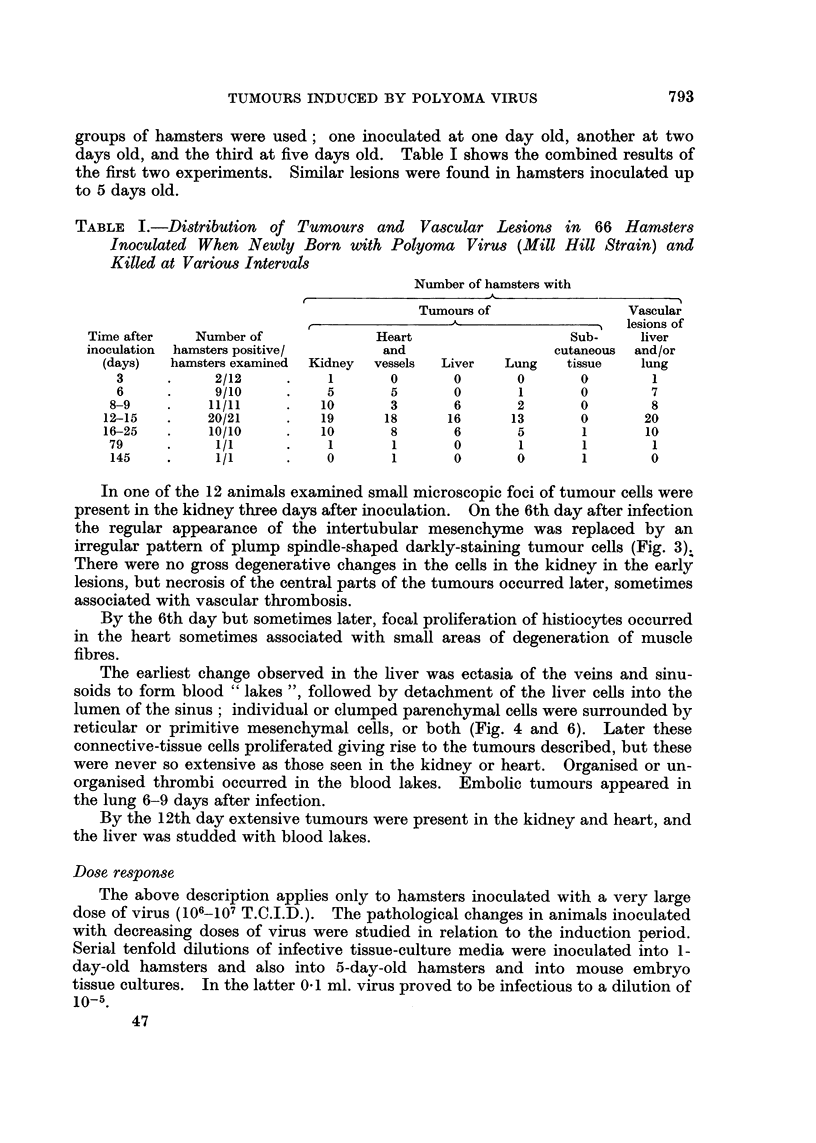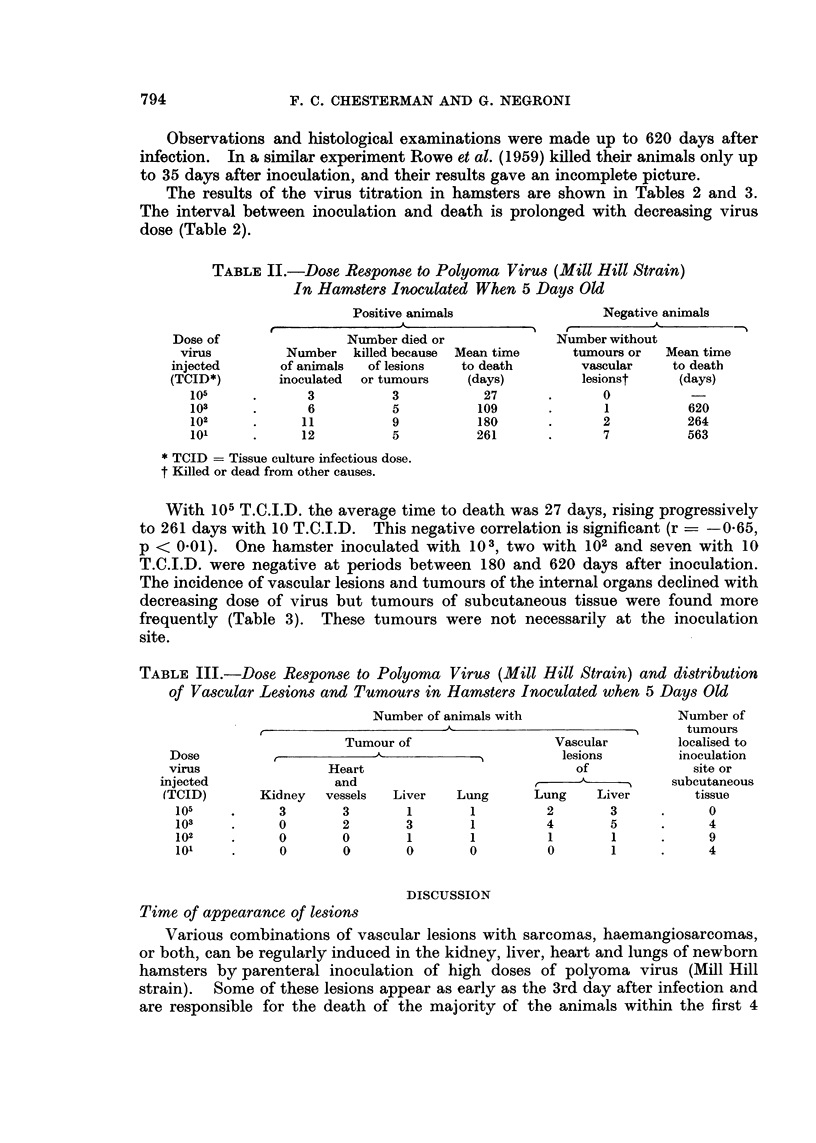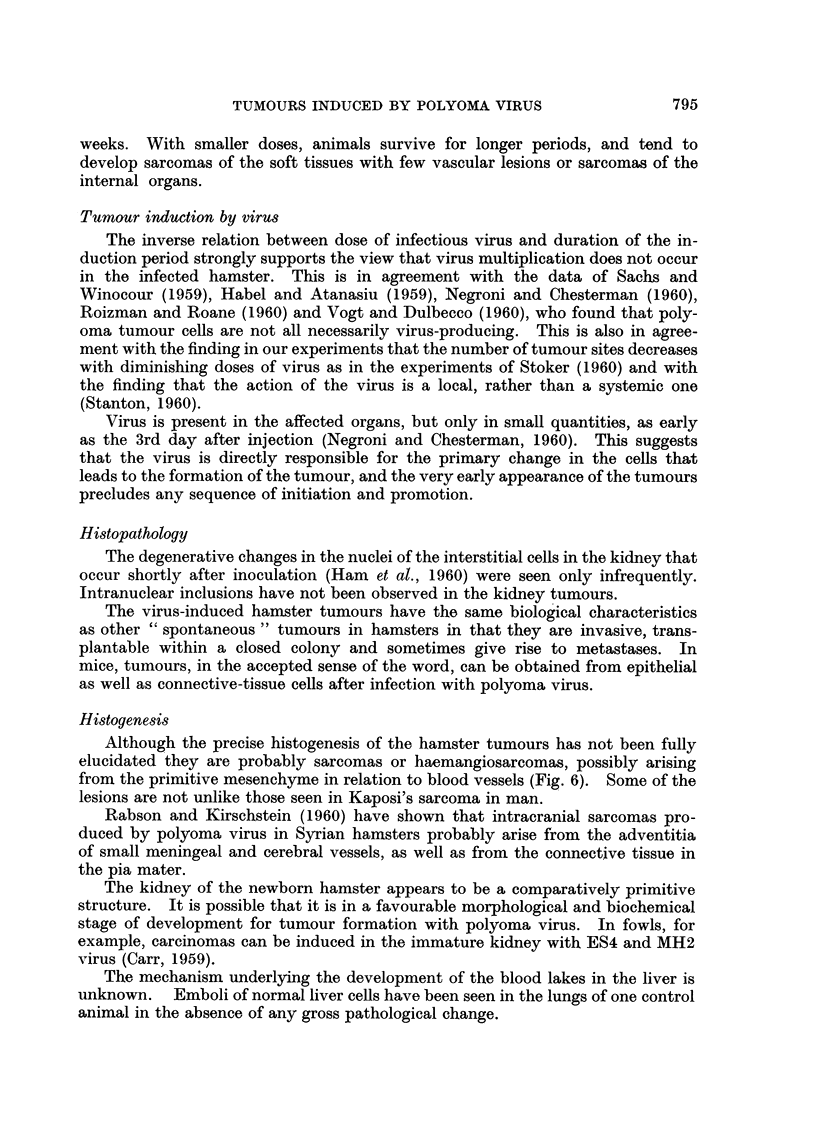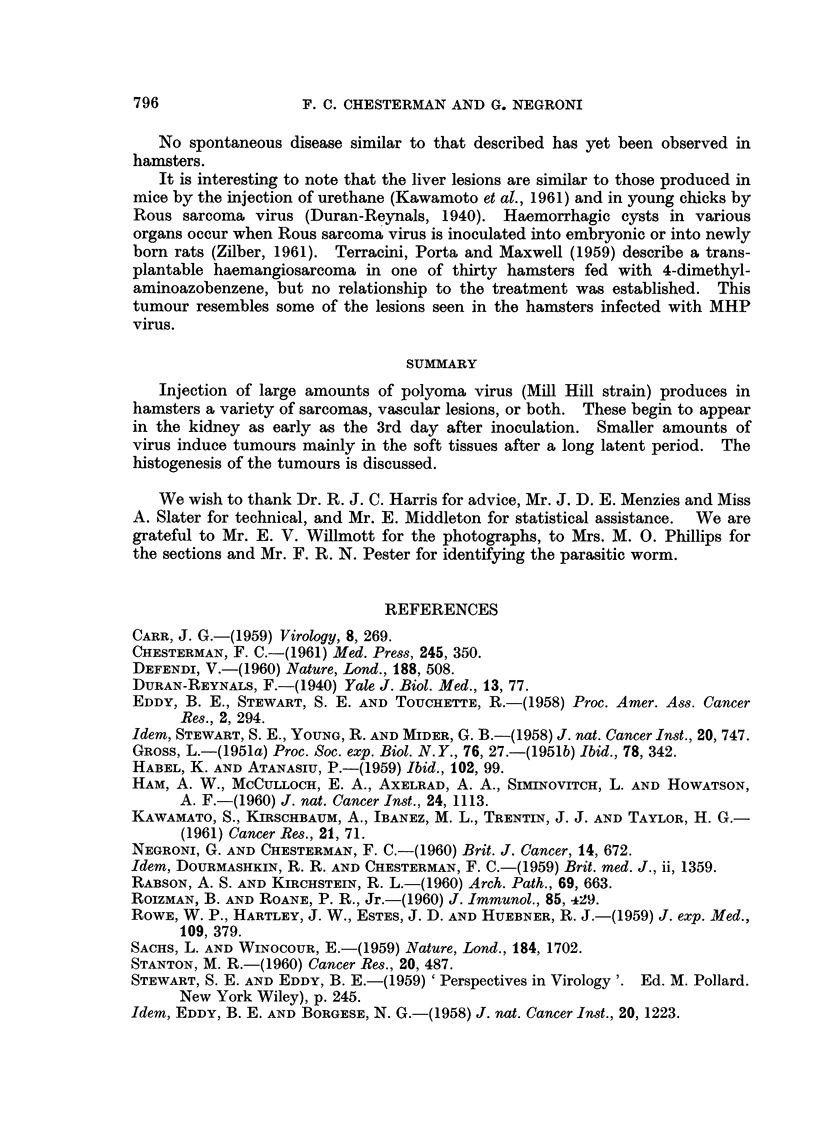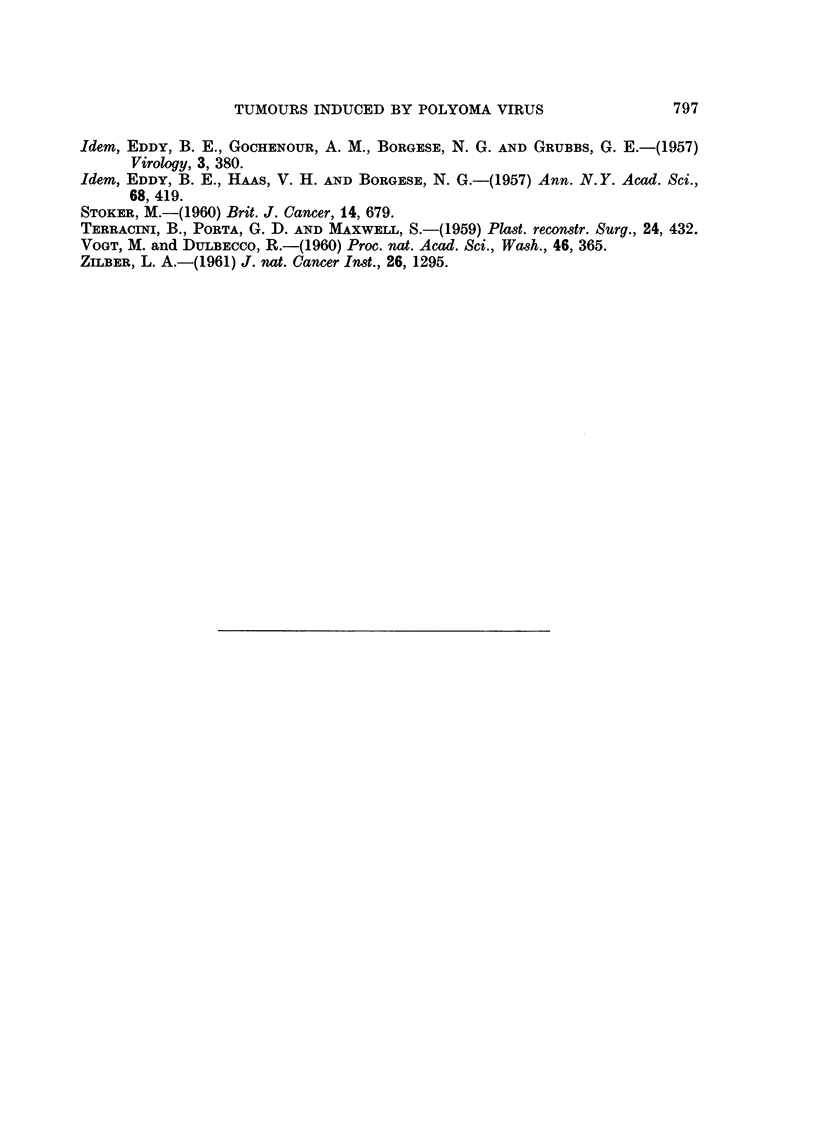# Tumours and other Lesions Induced in Golden Hamsters by a Polyoma Virus (Mill Hill Strain): Induction Time and Dose Response

**DOI:** 10.1038/bjc.1961.89

**Published:** 1961-12

**Authors:** F. C. Chesterman, G. Negroni

## Abstract

**Images:**


### TUMOURS AND OTHER LESIONS INDUCED IN GOLDEN HAMSTERS BY A POLYOMA VIRUS (MILL HILL STRAIN): INDUCTION TIME AND DOSE RESPONSE

F. C. CHESTERMAN AND G. NEGRONI

*From the Division of Experimental Biology and Virology, Imperial Cancer Research Fund, Mill Hill, London, N.W.7.*

Received for publication November 1, 1961

IN 1957 Stewart, Eddy and their colleagues isolated polyoma virus (SEP) in tissue culture from virus induced parotid tumours and leukaemias of mice (Stewart, Eddy, Gochenour, *et al.*, 1957 ; Stewart, Eddy, Haas and Borgese, 1957 ; Eddy, Stewart and Touchette, 1958 ; Eddy, Stewart, Young and Mider, 1958 ; Stewart, Eddy and Borgese, 1958 ; Stewart and Eddy, 1959).

In 1959 we isolated a similar polyoma virus (Mill Hill strain) from the spleen of a leukaemic AK mouse (Negroni, Dourmashkin and Chesterman, 1959), and subsequently from the spleen of a C3H mouse with virus-induced leukaemia by the technique of Gross (1951*a* and *b*). The properties of the Mill Hill strain polyoma virus (MHP) are similar to those of the SEP polyoma virus.

When the MHP virus was inoculated into hamsters 1–5 days old it produced vascular lesions, sarcomas or both in the kidney, liver, heart, lung and other tissues. The tumours were already extensive 2–3 weeks after inoculation of the virus. It was therefore important to determine the first appearance of tumour cells and their distribution in different organs. This will be described in the present paper together with the pathological changes obtained by varying the dose of infectious virus inoculated into the hamsters.

### MATERIAL AND METHODS

*Hamsters.*—Golden hamsters have been bred in a closed colony at Mill Hill for the last 8 years, but not by brother-sister mating. Those used were kept in galvanised wire cages and fed on a pellet diet (41*b*) mixed with puppy dog biscuits (Saval) and given water and greenstuff *ad libitum.* Infected litters were examined once or twice daily for clinical assessment, and to remove the dying or dead before they were eaten.

*Virus.*—The virus preparations were derived : (1) from the spleen of a male AK mouse with spontaneous lymphocytic leukaemia : (2) from the spleen of a C3H mouse with virus-induced leukaemia by the technique of Gross (1951*a* and *b*) : and (3) from polyoma-induced tumours of hamsters.

In all cases the virus was isolated by inoculating mouse embryo monolayer tissue cultures with cell suspensions, and the virus was passed serially in similar cultures of mouse embryo tissues. The medium from these cultures contained $10^6$–$10^7$ tissue-culture infectious doses (T.C.I.D.) per ml., and was used fresh or frozen, as infecting virus suspension for the experiments. This virus suspension was centrifuged, with or without subsequent filtration through bacteria-tight sintered glass filters, and single doses of 0·05 ml. were inoculated intraperitoneally or subcutaneously into baby hamsters 1–5 days old.

Control animals comprised those inoculated with (1) media or media and cells from tissue cultures of normal mouse embryo cells, and (2) cell suspensions of normal hamster kidney.

*Histology.*—Slices of the organs were fixed in neutral formalin or Zenker's solution, embedded in paraffin and stained with Ehrlich's haematoxylin and eosin.

Initially almost all organs were sectioned and studied, but detailed microscopical examination was later confined to the kidney, liver, heart and lung, and to macroscopic tumours arising at other sites.

### RESULTS

### *Post mortem examination*

MHP virus produces tumours or vascular lesions or both in over 90 per cent of hamsters inoculated parenterally with $10^6$–$10^7$ T.C.I.D. Most of the hamsters died between 12 and 31 days after inoculation.

At autopsy the livers showed many circumscribed blood-red spots up to several millimeters in diameter, flush with the surface or projecting above it. The intermediate liver was normal or pale. On cutting the liver, blood-stained fluid escaped from the cysts, giving it a honeycomb appearance (Fig. 1). Some of the larger cysts contained ante mortem thrombi. In females minute haemorrhagic cysts could sometimes be seen in the uteri and ovaries.

The kidneys were enlarged and pale, and often contained several white nodules projecting above the surface. Less frequently there were small subcapsular haemorrhages. In older lesions, the cut surface showed firm white tissue replacing the kidney, but only partially involving a thin outer rim of the cortex and sparing the papilla which is relatively long in the hamster (Chesterman, 1961). The heart often showed tumour nodules in the auricular and ventricular walls (Fig. 2). These tumours projected into the heart chamber, or into the mediastinum with only a thin pedicle attached to the heart. Similar tumours adhered to the outer coats of the veins near their entrance to the heart. In moribund animals bloody fluid could sometimes be observed around the mouth and nose, and at necropsy blood was present in the trachea and main bronchi. The lungs appeared normal or showed multiple small red spots, sometimes projecting above the pleural surface. In some animals surviving for more than two weeks small grey tumours were also present, either separately or in the centre of some of the red lesions. Death was often associated with haemorrhage from rupture of one or more haemorrhagic liver lesions, extensive sarcomatous involvement of the kidneys, or both.

### *Histology*

There were two main groups of lesions : (1) tumours, (2) vascular lesions.

*Tumours.*—Morphologically the tumours were spindle-cell sarcomas composed of darkly-stained cells varying in size and showing nuclear irregularity. In the kidney they appeared first in the cortico-medullary junction and later involved the inner cortex and outer medulla destroying the kidney tubules (Fig. 3). Multiple nodules could sometimes be seen in the outer cortex. Morphologically similar sarcomas were present in the heart muscle or valves. As these tumours increased in size the centre underwent necrosis. Similar sarcomas were sometimes present in the liver, particularly near the hilum where the inferior vena cava passes through the diaphragm, in the lungs, and in one instance attached to the outer bowel wall, as described by Stoker (1960) for the B.P. (Toronto strain) virus.

In three cases only were subcutaneous sarcomas seen. One about 3 mm. in diameter occurred near the external auditory meatus of a hamster dying from internal lesions 20 days after inoculation. Two more were found in animals dying on the 79th and 145th day after inoculation. Subcutaneous sarcomas were more frequent when lower doses of virus were used (*vide infra*) or after a long latent period when adult animals were inoculated with high doses of virus. This latter finding is similar to the results of Defendi (1960).

Areas of connective-tissue mesenchymal hyperplasia were sometimes present in the testis, adrenal cortex, ovary and meninges.

Another type of tumour formed by proliferation of cells lining the blood lakes and resembling an angiosarcoma was present in the liver and sometimes as emboli in the lungs.

*Vascular lesions.*—These occurred frequently in the liver as dilatation of the sinusoids and ends of the interlobular portal and intralobular central veins, forming blood lakes with disruption and loss of adhesion of liver cells so that the cells formed clumps within the lakes (Fig. 4). Focal areas of necrosis unaccompanied by surrounding inflammatory cell infiltration but sometimes associated with haemorrhage or small intrasinusoidal thrombi were also seen. Occasionally fragments of the larval stage of *Cysticercus fasciolaris* were present.

In the lungs there were areas of intra-alveolar or interstitial haemorrhage, or both, with consolidation and vascular dilatation similar to that seen in the liver but usually not so extensive. The walls of some of the distended vessels appeared to contain dissecting aneurysms.

Emboli in the lung were either " normal " liver cells or clumps of sarcoma or angiosarcoma cells (Fig. 5). In a few animals vascular dilatation and sometimes haemorrhage were present in the brain, ovary, uterus, bowel and subcutaneous tissues.

*Induction time*

In order to study the development of the tumours and vascular lesions, groups of 2–5 animals were killed at 2–3 day intervals after virus inoculation. Three

---

EXPLANATION OF PLATES

Fig. 1.—Liver of female hamster aged 22 days inoculated with MHP virus when 2 days old. Honeycomb appearance. ×6·5.

Fig. 2.—Heart of male hamster aged 27 days inoculated with MHP virus when 2 days old. Multiple nodules replace the ventricular walls. ×6.

Fig. 3.—Kidney of male hamster aged 7 days, 6 days after inoculation with MHP virus. Early tumour growth. ×315.

Fig. 4.—Liver of male hamster inoculated with MHP virus when 2 days old. Clumps of liver cells lie in a blood lake continuous with a central vein. ×95.

Fig. 5.—Lung from male hamster aged 27 days, inoculated with MHP virus when 2 days old. Top right, a clump of normal liver cells ; botton left, a clump of tumour cells. ×650.

Fig. 6.—Liver of male hamster aged 27 days inoculated with MHP virus when 2 days old. Proliferation of mesenchyme cells between parenchymal liver cells and the wall of the sinusoids. ×650.

All sections stained H. and E.

1

2

3

Chesterman and Negroni.

4

5

6

groups of hamsters were used ; one inoculated at one day old, another at two days old, and the third at five days old. Table I shows the combined results of the first two experiments. Similar lesions were found in hamsters inoculated up to 5 days old.

TABLE I.—*Distribution of Tumours and Vascular Lesions in 66 Hamsters Inoculated When Newly Born with Polyoma Virus (Mill Hill Strain) and Killed at Various Intervals*

| | | Number of hamsters with | | | | | |
|---|---|---|---|---|---|---|---|
| | | Tumours of | | | | | Vascular lesions of |
| Time after inoculation (days) | Number of hamsters positive/ hamsters examined | Kidney | Heart and vessels | Liver | Lung | Sub-cutaneous tissue | liver and/or lung |
| 3 | 2/12 | 1 | 0 | 0 | 0 | 0 | 1 |
| 6 | 9/10 | 5 | 5 | 0 | 1 | 0 | 7 |
| 8–9 | 11/11 | 10 | 3 | 6 | 2 | 0 | 8 |
| 12–15 | 20/21 | 19 | 18 | 16 | 13 | 0 | 20 |
| 16–25 | 10/10 | 10 | 8 | 6 | 5 | 1 | 10 |
| 79 | 1/1 | 1 | 1 | 0 | 1 | 1 | 1 |
| 145 | 1/1 | 0 | 1 | 0 | 0 | 1 | 0 |

In one of the 12 animals examined small microscopic foci of tumour cells were present in the kidney three days after inoculation. On the 6th day after infection the regular appearance of the intertubular mesenchyme was replaced by an irregular pattern of plump spindle-shaped darkly-staining tumour cells (Fig. 3). There were no gross degenerative changes in the cells in the kidney in the early lesions, but necrosis of the central parts of the tumours occurred later, sometimes associated with vascular thrombosis.

By the 6th day but sometimes later, focal proliferation of histiocytes occurred in the heart sometimes associated with small areas of degeneration of muscle fibres.

The earliest change observed in the liver was ectasia of the veins and sinusoids to form blood " lakes ", followed by detachment of the liver cells into the lumen of the sinus ; individual or clumped parenchymal cells were surrounded by reticular or primitive mesenchymal cells, or both (Fig. 4 and 6). Later these connective-tissue cells proliferated giving rise to the tumours described, but these were never so extensive as those seen in the kidney or heart. Organised or unorganised thrombi occurred in the blood lakes. Embolic tumours appeared in the lung 6–9 days after infection.

By the 12th day extensive tumours were present in the kidney and heart, and the liver was studded with blood lakes.

*Dose response*

The above description applies only to hamsters inoculated with a very large dose of virus ($10^6$–$10^7$ T.C.I.D.). The pathological changes in animals inoculated with decreasing doses of virus were studied in relation to the induction period. Serial tenfold dilutions of infective tissue-culture media were inoculated into 1-day-old hamsters and also into 5-day-old hamsters and into mouse embryo tissue cultures. In the latter 0·1 ml. virus proved to be infectious to a dilution of $10^{-5}$.

Observations and histological examinations were made up to 620 days after infection. In a similar experiment Rowe *et al.* (1959) killed their animals only up to 35 days after inoculation, and their results gave an incomplete picture.

The results of the virus titration in hamsters are shown in Tables 2 and 3. The interval between inoculation and death is prolonged with decreasing virus dose (Table 2).

TABLE II.—*Dose Response to Polyoma Virus (Mill Hill Strain) In Hamsters Inoculated When 5 Days Old*

| | Positive animals | | | Negative animals | |
|---|---|---|---|---|---|
| Dose of virus injected (TCID*) | Number of animals inoculated | Number died or killed because of lesions or tumours | Mean time to death (days) | Number without tumours or vascular lesions† | Mean time to death (days) |
| $10^5$ | 3 | 3 | 27 | 0 | — |
| $10^3$ | 6 | 5 | 109 | 1 | 620 |
| $10^2$ | 11 | 9 | 180 | 2 | 264 |
| $10^1$ | 12 | 5 | 261 | 7 | 563 |

* TCID = Tissue culture infectious dose.
† Killed or dead from other causes.

With $10^5$ T.C.I.D. the average time to death was 27 days, rising progressively to 261 days with 10 T.C.I.D. This negative correlation is significant ($r = -0{\cdot}65$, $p < 0{\cdot}01$). One hamster inoculated with $10^3$, two with $10^2$ and seven with 10 T.C.I.D. were negative at periods between 180 and 620 days after inoculation. The incidence of vascular lesions and tumours of the internal organs declined with decreasing dose of virus but tumours of subcutaneous tissue were found more frequently (Table 3). These tumours were not necessarily at the inoculation site.

TABLE III.—*Dose Response to Polyoma Virus (Mill Hill Strain) and distribution of Vascular Lesions and Tumours in Hamsters Inoculated when 5 Days Old*

| | Number of animals with | | | | | | |
|---|---|---|---|---|---|---|---|
| | Tumour of | | | | Vascular lesions of | | |
| Dose virus injected (TCID) | Kidney | Heart and vessels | Liver | Lung | Lung | Liver | Number of tumours localised to inoculation site or subcutaneous tissue |
| $10^5$ | 3 | 3 | 1 | 1 | 2 | 3 | 0 |
| $10^3$ | 0 | 2 | 3 | 1 | 4 | 5 | 4 |
| $10^2$ | 0 | 0 | 1 | 1 | 1 | 1 | 9 |
| $10^1$ | 0 | 0 | 0 | 0 | 0 | 1 | 4 |

### DISCUSSION

### *Time of appearance of lesions*

Various combinations of vascular lesions with sarcomas, haemangiosarcomas, or both, can be regularly induced in the kidney, liver, heart and lungs of newborn hamsters by parenteral inoculation of high doses of polyoma virus (Mill Hill strain). Some of these lesions appear as early as the 3rd day after infection and are responsible for the death of the majority of the animals within the first 4

weeks. With smaller doses, animals survive for longer periods, and tend to develop sarcomas of the soft tissues with few vascular lesions or sarcomas of the internal organs.

*Tumour induction by virus*

The inverse relation between dose of infectious virus and duration of the induction period strongly supports the view that virus multiplication does not occur in the infected hamster. This is in agreement with the data of Sachs and Winocour (1959), Habel and Atanasiu (1959), Negroni and Chesterman (1960), Roizman and Roane (1960) and Vogt and Dulbecco (1960), who found that polyoma tumour cells are not all necessarily virus-producing. This is also in agreement with the finding in our experiments that the number of tumour sites decreases with diminishing doses of virus as in the experiments of Stoker (1960) and with the finding that the action of the virus is a local, rather than a systemic one (Stanton, 1960).

Virus is present in the affected organs, but only in small quantities, as early as the 3rd day after injection (Negroni and Chesterman, 1960). This suggests that the virus is directly responsible for the primary change in the cells that leads to the formation of the tumour, and the very early appearance of the tumours precludes any sequence of initiation and promotion.

*Histopathology*

The degenerative changes in the nuclei of the interstitial cells in the kidney that occur shortly after inoculation (Ham *et al.*, 1960) were seen only infrequently. Intranuclear inclusions have not been observed in the kidney tumours.

The virus-induced hamster tumours have the same biological characteristics as other " spontaneous " tumours in hamsters in that they are invasive, transplantable within a closed colony and sometimes give rise to metastases. In mice, tumours, in the accepted sense of the word, can be obtained from epithelial as well as connective-tissue cells after infection with polyoma virus.

*Histogenesis*

Although the precise histogenesis of the hamster tumours has not been fully elucidated they are probably sarcomas or haemangiosarcomas, possibly arising from the primitive mesenchyme in relation to blood vessels (Fig. 6). Some of the lesions are not unlike those seen in Kaposi's sarcoma in man.

Rabson and Kirschstein (1960) have shown that intracranial sarcomas produced by polyoma virus in Syrian hamsters probably arise from the adventitia of small meningeal and cerebral vessels, as well as from the connective tissue in the pia mater.

The kidney of the newborn hamster appears to be a comparatively primitive structure. It is possible that it is in a favourable morphological and biochemical stage of development for tumour formation with polyoma virus. In fowls, for example, carcinomas can be induced in the immature kidney with ES4 and MH2 virus (Carr, 1959).

The mechanism underlying the development of the blood lakes in the liver is unknown. Emboli of normal liver cells have been seen in the lungs of one control animal in the absence of any gross pathological change.

No spontaneous disease similar to that described has yet been observed in hamsters.

It is interesting to note that the liver lesions are similar to those produced in mice by the injection of urethane (Kawamoto *et al.*, 1961) and in young chicks by Rous sarcoma virus (Duran-Reynals, 1940). Haemorrhagic cysts in various organs occur when Rous sarcoma virus is inoculated into embryonic or into newly born rats (Zilber, 1961). Terracini, Porta and Maxwell (1959) describe a transplantable haemangiosarcoma in one of thirty hamsters fed with 4-dimethylaminoazobenzene, but no relationship to the treatment was established. This tumour resembles some of the lesions seen in the hamsters infected with MHP virus.

### SUMMARY

Injection of large amounts of polyoma virus (Mill Hill strain) produces in hamsters a variety of sarcomas, vascular lesions, or both. These begin to appear in the kidney as early as the 3rd day after inoculation. Smaller amounts of virus induce tumours mainly in the soft tissues after a long latent period. The histogenesis of the tumours is discussed.

We wish to thank Dr. R. J. C. Harris for advice, Mr. J. D. E. Menzies and Miss A. Slater for technical, and Mr. E. Middleton for statistical assistance. We are grateful to Mr. E. V. Willmott for the photographs, to Mrs. M. O. Phillips for the sections and Mr. F. R. N. Pester for identifying the parasitic worm.

### REFERENCES

Carr, J. G.—(1959) *Virology*, **8**, 269.
Chesterman, F. C.—(1961) *Med. Press*, **245**, 350.
Defendi, V.—(1960) *Nature, Lond.*, **188**, 508.
Duran-Reynals, F.—(1940) *Yale J. Biol. Med.*, **13**, 77.
Eddy, B. E., Stewart, S. E. and Touchette, R.—(1958) *Proc. Amer. Ass. Cancer Res.*, **2**, 294.
*Idem*, Stewart, S. E., Young, R. and Mider, G. B.—(1958) *J. nat. Cancer Inst.*, **20**, 747.
Gross, L.—(1951*a*) *Proc. Soc. exp. Biol. N.Y.*, **76**, 27.—(1951*b*) *Ibid.*, **78**, 342.
Habel, K. and Atanasiu, P.—(1959) *Ibid.*, **102**, 99.
Ham, A. W., McCulloch, E. A., Axelrad, A. A., Siminovitch, L. and Howatson, A. F.—(1960) *J. nat. Cancer Inst.*, **24**, 1113.
Kawamato, S., Kirschbaum, A., Ibanez, M. L., Trentin, J. J. and Taylor, H. G.—(1961) *Cancer Res.*, **21**, 71.
Negroni, G. and Chesterman, F. C.—(1960) *Brit. J. Cancer*, **14**, 672.
*Idem*, Dourmashkin, R. R. and Chesterman, F. C.—(1959) *Brit. med. J.*, ii, 1359.
Rabson, A. S. and Kirchstein, R. L.—(1960) *Arch. Path.*, **69**, 663.
Roizman, B. and Roane, P. R., Jr.—(1960) *J. Immunol.*, **85**, 429.
Rowe, W. P., Hartley, J. W., Estes, J. D. and Huebner, R. J.—(1959) *J. exp. Med.*, **109**, 379.
Sachs, L. and Winocour, E.—(1959) *Nature, Lond.*, **184**, 1702.
Stanton, M. R.—(1960) *Cancer Res.*, **20**, 487.
Stewart, S. E. and Eddy, B. E.—(1959) ' Perspectives in Virology '. Ed. M. Pollard. New York Wiley), p. 245.
*Idem*, Eddy, B. E. and Borgese, N. G.—(1958) *J. nat. Cancer Inst.*, **20**, 1223.

*Idem*, EDDY, B. E., GOCHENOUR, A. M., BORGESE, N. G. AND GRUBBS, G. E.—(1957) *Virology*, **3**, 380.
*Idem*, EDDY, B. E., HAAS, V. H. AND BORGESE, N. G.—(1957) *Ann. N.Y. Acad. Sci.*, **68**, 419.
STOKER, M.—(1960) *Brit. J. Cancer*, **14**, 679.
TERRACINI, B., PORTA, G. D. AND MAXWELL, S.—(1959) *Plast. reconstr. Surg.*, **24**, 432.
VOGT, M. and DULBECCO, R.—(1960) *Proc. nat. Acad. Sci., Wash.*, **46**, 365.
ZILBER, L. A.—(1961) *J. nat. Cancer Inst.*, **26**, 1295.